# Characteristics of Driving Risk Checklist‐25 among community‐dwelling older adults with mild cognitive impairment

**DOI:** 10.1111/ggi.70127

**Published:** 2025-08-13

**Authors:** Ayuto Kodama, Takuji Nakamura, Hideyuki Azuma, Yukiko Mouri, Yuji Tanaka, Hidenori Tochigi, Hidetaka Ota

**Affiliations:** ^1^ Advanced Research Center for Geriatrics and Gerontology Akita University Akita Japan; ^2^ Department of Occupational Therapy Graduate School of Medicine, Akita University Akita Japan; ^3^ TACT Corporation Yokohama Japan; ^4^ The Japan Research Institute General Incorporated Association Tokyo Japan; ^5^ Pacific Consultants Corporation

**Keywords:** drive, gait speed, geriatrics, mild cognitive impairment, region

## Abstract

**Aim:**

With the rapid increase of aging societies worldwide, ensuring road safety among older adults has become a critical public health concern. This study aimed to assess driving risks among community‐dwelling older adults with mild cognitive impairment (MCI).

**Methods:**

A total of 387 participants aged 65 years and older were recruited from Akita Prefecture, Japan. Physical functions, such as walking speed and grip strength, cognitive functions, and depressive symptoms were evaluated alongside driving behaviors. Participants were classified into MCI‐positive and MCI‐negative groups based on cognitive assessments.

**Results:**

The results demonstrated significant differences in driving risk, physical performance, and cognitive abilities between the two groups. MCI‐positive individuals exhibited higher driving risks and poorer functional performance. Receiver operating characteristic (ROC) curve analysis identified a cutoff score of 5.5 on the Driving Risk Checklist‐25 (DRCL‐25), with a sensitivity of 45.5% and specificity of 89.7% for identifying MCI.

**Conclusions:**

These findings underscore the importance of integrating physical and cognitive assessments to enhance the accuracy of driving risk evaluations. The DRCL‐25 shows promise as a detection tool for MCI, facilitating the development of interventions aimed at maintaining mobility and improving road safety among older drivers. Future research should focus on refining the checklist and evaluating its applicability across diverse populations and settings. **Geriatr Gerontol Int 2025; 25: 1194–1199**.

## Graphical abstract

This study examined driving risk in older adults with mild cognitive impairment (MCI) using the Driving Risk Checklist‐25 (DRCL‐25). MCI‐positive individuals showed a significantly higher driving risk and poorer physical and cognitive performance. A DRCL‐25 cutoff score of 5.5 effectively identified MCI, supporting its utility as a screening tool.

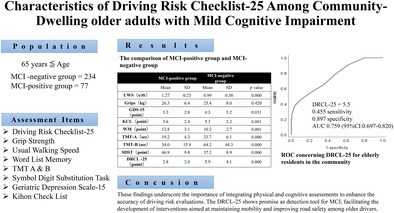

## Introduction

With the progression of aging societies, driving among older adults has become a significant social concern. Cognitive decline, in particular, has garnered attention for its association with an increased risk of traffic accidents.^1^, ^2^ Mild cognitive impairment (MCI) represents a high‐risk state for progression to dementia, highlighting the importance of early detection at this stage.^3^ Recent studies have demonstrated the utility of tools measuring visual attention, spatial cognition, and executive functions to evaluate the effects of cognitive decline on driving ability.^4^, ^5^

In Japan, the “30‐item checklist for early detection of cognitive impairment while driving,” developed by the Elderly Drivers Safety Support Research Association, has been widely used to identify early‐stage cognitive impairment. This tool assesses specific behaviors and judgment abilities related to driving, thereby supporting safer driving practices among older adults.^1^ A simplified version, the “Driving Risk Checklist‐25” (DRCL‐25), was subsequently developed to streamline the evaluation of driving behaviors and to clarify the relationship between cognitive function and driving risk.^6^ The DRCL‐25 is based on previous findings by geriatricians and road traffic experts (data unpublished), and each of the 30 items in the DRCL was further weighted and simplified into more important and influential items for older drivers.

Furthermore, studies investigating the interplay between physical and cognitive functions have suggested that declines in gait speed and grip strength may serve as early indicators of cognitive impairment.^7^, ^8^ By integrating these evaluation metrics, a multifaceted assessment of driving ability among older adults can be achieved, potentially preventing traffic accidents and enhancing driving support.^9^ This underscores the importance of evaluating motor function alongside cognitive abilities, as the two domains are closely interconnected in tasks requiring complex decision‐making, such as driving.^10^

Recent advancements in technology, including wearable devices and motion analysis systems, provide new opportunities for continuous and precise monitoring of gait and grip strength in real‐world settings. These technologies enable the early detection of subtle functional declines that might be overlooked during traditional clinical assessments. Additionally, combining cognitive tests – such as those assessing reaction time and attention span – with physical performance metrics offers a comprehensive framework for evaluating driving risks. Such an approach aids in identifying individuals at higher risk of accidents and facilitates tailored interventions, including physical rehabilitation programs, cognitive training, or adaptive driving support systems. These interventions can help maintain mobility and independence among older adults while addressing public safety concerns associated with aging drivers.

The purpose of this study is to assess driving risks among community‐dwelling older adults with MCI.

## Methods

### Participants

The participants in this study were 387 persons selected from the population of three municipalities in Akita Prefecture aged 65 years or older through public recruitment in each city (mean age: 76.6 ± 5.6 years). The study was conducted from mid‐November 2023 to the end of August 2024. Participants were excluded if they had severe cardiovascular or central nervous system diseases, were certified for long‐term care, or had missing data in any evaluation or measurement items. The exclusion criteria also included the need for support or care as certified by the Japanese public long‐term care insurance system owing to disability. The study was approved by the Ethics Committee of the Faculty of Medicine, Akita University (approval no. 273) and was performed in accordance with the Declaration of Helsinki II. Written informed consent was obtained from all participants.

### Assessment items

#### Basic demographics

Basic demographic information, including age, sex, height, weight, medication status, education, and the number of household members, was collected through interviews or measurements.

#### Physical function assessment

- Usual walking speed (UWS): UWS was measured using an Otassha 21 gait analyzer (Yagami Co., Ltd, Nagoya, Japan). The measurement was performed on a 5‐m walkway with an additional 1‐m preparation area at both ends to ensure smooth acceleration and deceleration.
- Grip strength (GS): GS was measured using a Smedley‐type hand dynamometer (T.K.K. 5401, Takei Scientific Instruments, Tokyo, Japan). The participants were instructed to stand in an upright position during the measurement.

#### Cognitive function assessment

Cognitive function was evaluated using the National Center for Geriatrics and Gerontology Functional Assessment Tool (NCGG‐FAT), a validated, tablet‐based system designed for objective cognitive testing. The NCGG‐FAT comprises the following four assessments.

- Word List Memory (WM): Memory ability was assessed through immediate and delayed recall tasks. The scores of the two tasks were summed to calculate the total WM score.
- Trail‐Making Tests A and B (TMT‐A and TMT‐B): TMT‐A required participants to select numbers in sequential order, while TMT‐B required alternating between numbers and characters. The time taken to complete each task was recorded.
- Symbol Digit Substitution Task (SDST): Participants matched symbols with their corresponding digits within 90 s. The total number of correct responses was recorded.

According to Petersen's report, in which individuals who showed cognitive impairment but were independent in activities of daily living were defined as having MCI, we applied MCI classification according to the cutoff point of NCGG‐FAT.^12^ For all cognitive subtests of NCGG‐FAT, the standardized threshold in each corresponding domain for defining impairment in Japanese population‐based cohorts consisting of older community‐dwellers is a score of more than 1.5 standard deviations (SD) below the age‐ and education‐specific mean.^1^ Participants with at least one positive result in these tests were classified as MCI‐positive, while the remaining participants were classified as MCI‐negative.

#### Depression assessment

Depressive tendencies were evaluated using the Geriatric Depression Scale‐15 (GDS‐15), a 15‐item questionnaire with a dichotomous (Yes/No) response format.^11^ This format facilitates ease of administration. Conventionally, five or more positive responses indicate mild depression, while 10 or more positive responses suggest moderate to severe depression. Clinical evaluation is recommended to confirm a diagnosis in such cases.

#### Functional ability assessment

The Kihon Checklist (KCL) was employed to evaluate participants' functional status.^13^ The KCL consists of 25 items (Q1–Q25), each assessing specific difficulties in daily activities. A higher total score indicates a greater risk of requiring support in daily life.

#### Driving Risk Checklist‐25 (DRCL‐25)

The DRCL‐25 was developed from the original 30‐item checklist and includes 25 items that assess cognitive and behavioral risks related to driving. A higher number of applicable items corresponds to a higher driving‐related risk. (Refer to Table S1 for details).

### Statistical analysis

An independent *t*‐test was conducted to compare basic attributes (age, height, weight, medication status, educational history, and number of household members), UWS, GS, WM, TMT‐A and TMT‐B, SDST, GDS‐15, KCL, and DRCL‐25 between the MCI‐positive and MCI‐negative groups. In addition, a *χ* ^2^ test of independence was performed to examine differences in sex, medical history, and the subitems of the DRCL‐25 between the two groups.

To determine the cutoff value of the DRCL‐25 for differentiating MCI, a receiver operating characteristic (ROC) curve analysis was conducted, using the MCI‐positive and MCI‐negative groups as the state variables. The ROC curve assessed the performance of the regression model by calculating the area under the curve (AUC), sensitivity, and specificity. The optimal cutoff value was determined as the point where the Youden index (sensitivity + specificity − 1) was maximized.

All statistical analyses were performed using SPSS version 27.0 for Windows (SPSS Inc., Chicago, IL, USA). The significance level was set at 5%.

## Results

The final analysis included 311 participants (mean age: 76.2 ± 5.6 years; 159 males and 152 females), with 234 individuals in the MCI‐negative group and 77 in the MCI‐positive group. Table 1 summarizes the basic attributes of the two groups. Compared with the MCI‐positive group, the MCI‐negative group was significantly younger (*P* < 0.05), took fewer medications (*P* < 0.05), and had a higher level of education (*P* < 0.05).

**Table 1:** Characteristics of the participants

*n*	MCI‐negative group		MCI‐positive group		
	234		77		
	Mean	SD	Mean	SD	*P*‐value
Age (years old)	74.4	5.1	75.9	5.1	0.002**
Sex (% female)	51.7		49.4		0.719
Height (cm)	155.3	8.0	154.4	10.4	0.210
Weight (kg)	56.9	8.6	58.4	10.4	0.621
Number of household members (people)	2.4	1.4	2.6	1.4	0.409
Medication (n)	2.5	2.6	3.2	2.5	0.036*
Education (years)	12.0	2.0	11.7	1.9	0.019*

*P < 0.05; **P < 0.01. Age, Education, Height, Weight, Number of household members, Medication, Education show the *P*‐value of the unpaired *t*‐test. Sex shows the *P*‐value of the *χ* ^2^ test.

Table 2 compares the evaluation results across the two groups. Regarding physical function, the MCI‐negative group demonstrated significantly faster UWS compared with the MCI‐positive group (*P* < 0.01). In terms of cognitive function, the MCI‐negative group exhibited significantly better performance in memory (*P* < 0.05), attention (*P* < 0.01), executive function (*P* < 0.01), and processing speed (*P* < 0.01). Based on the GDS‐15, depressive tendencies were significantly lower in the MCI‐negative group than in the MCI‐positive group (*P* < 0.05). Results from the KCL indicated that the MCI‐negative group had significantly higher levels of daily functioning (*P* < 0.01). Furthermore, physical activity evaluations revealed that the MCI‐negative group had a significantly higher average daily step count compared with the MCI‐positive group (*P* < 0.05). Lastly, the DRCL‐25 scores indicated that the MCI‐negative group had a significantly lower driving risk than the MCI‐positive group (*P* < 0.01).

**Table 2:** Comparison of the MCI‐positive group and MCI‐negative group

	MCI‐negative group		MCI‐positive group		
	Mean	SD	Mean	SD	*P* value
UWS (s/m)	1.27	0.23	0.99	0.38	0.000**
Grip strength (kg)	26.3	6.4	25.4	8.0	0.420
GDS‐15 (points)	3.3	2.8	4.3	3.2	0.031*
KCL (points)	3.6	2.4	5.3	3.2	0.001**
WM (points)	12.8	3.1	10.2	2.7	0.001**
TMT‐A (s)	19.2	4.3	23.7	6.1	0.000**
TMT‐B (s)	34.0	15.9	64.2	44.3	0.000**
SDST (points)	46.9	9.8	37.2	8.9	0.000**
DRCL‐25 (points)	2.8	2.0	5.9	4.1	0.000**

*P < 0.05; **P < 0.01. The unpaired *t*‐test. DRCL‐25, Driving Risk Checklist‐25; GDS‐15, Geriatrics Depression Scale‐15; KCL, Kihon Checklist; SDST, Symbol Digit Substitution Task; TMT‐A, Trail‐Making Test‐A; TMT‐B, Trail Making Test‐B; UWS, usual walking speed; WM, word list memory.

Table 3 presents a comparison of DRCL‐25 subitems between the two groups. Participants in the MCI‐negative group had significantly more difficulties and negative experiences than those in the MCI‐positive group in the following areas. Feeling that more effort is required to turn the steering wheel in parking lots (*P* < 0.01). Relying on mirrors instead of direct visual checks during lane changes or turns (*P* < 0.01). Becoming less concerned about car cleanliness or damage (*P* < 0.01). Gradual reduction in driving for leisure (*P* < 0.01). Feeling more fatigued after driving (*P* < 0.05). Perceiving slower braking responses (*P* < 0.01). Experiencing frequent startling or near‐miss incidents while driving (*P* < 0.05). Forgetting where keys or licenses were placed more often (*P* < 0.01). Difficulty merging or changing lanes on highways or bypasses (*P* < 0.01). Trouble parking within garage or parking lot boundaries (*P* < 0.01). Increased difficulty turning right at large intersections (*P* < 0.01). Forgetting to use turn signals during turns or lane changes (*P* < 0.01). Avoiding conversations with passengers while driving (*P* < 0.01). Being told by usual passengers that their driving has become rough (*P* < 0.01). Experiencing frequent insomnia or shallow sleep (*P* < 0.05). Feeling startled by sudden appearances of pedestrians or bicycles during turns (*P* < 0.01). Making navigation errors on familiar roads (*P* < 0.01). Forgetting intended destinations while driving (*P* < 0.01). Instances of wrong‐way driving without realizing it (*P* < 0.01). Experiencing faintness or near‐blackouts while driving (*P* < 0.01).

**Table 3:** Comparison of DRCL‐25 subitems between the two groups

		MCI‐negative	MCI‐positive	
		%	Yes (%)	*P*‐value
1	When turning the steering wheel in parking lots, etc., I feel that more force is required than before.	5.5	22	0.000**
2	I feel that the timing of braking is slower than before.	3.4	26	0.000**
3	When changing lanes or making a right or left turn, they do not look straight ahead with their own eyes, but only at their mirrors.	12.4	31.2	0.000**
4	I often have near‐misses while driving.	18.4	36.4	0.001**
5	I don't care much about dirt and scratches on my car anymore, and I don't feel like cleaning it as much as before.	21.4	35.1	0.016*
6	The number of times I drive for pleasure, such as driving, is gradually decreasing.	44.4	64.9	0.002**
7	I forget where I put my keys, driver's license, etc., and have to look for them more often.	13.2	23.4	0.034*
8	Merging and changing lanes on highways and bypasses has become difficult.	25.2	44.2	0.002**
9	More often than not, I lose track of where I parked my car in large parking lots.	15	40.3	0.000**
10	I can no longer park well within the confines of my home's garage or parking lot.	13.2	22.1	0.063
11	After driving, I feel more tired than before.	20.9	39	0.002**
12	Sometimes I don't understand the meaning of a road sign that I used to understand.	1.3	15.6	0.000**
13	I have a hard time making right turns at large intersections.	5.6	22.1	0.000**
14	I am startled when I suddenly notice pedestrians and bicycles when turning right or left.	5.6	28.6	0.000**
15	I tend to forget to use my blinker when turning right or left or changing lanes.	0.4	5.2	0.004**
16	Even on familiar roads, I make more mistakes, such as where to turn.	3	9.1	0.025*
17	Talking to passengers while driving has become a hassle.	6.4	13	0.066
18	I forgot where I was going while driving.	1.7	5.2	0.094
19	My usual passengers tell me that my driving has become rough lately.	0.9	15.6	0.000**
20	Several times I have mis‐stomped on the brake and the gas pedal.	1.3	11.7	0.000**
21	There have been a few times when I have driven in the opposite direction without knowing it.	0	5.2	0.000**
22	I have forgotten to load passengers or luggage and departed.	2.1	5.2	0.165
23	I have felt faint or almost fainted while driving.	1.3	0	0.318
24	Often has trouble sleeping at night and sleeps poorly.	26.1	44.2	0.003**
25	I often take medications such as sleep aids to help me sleep.	13.7	28.6	0.003**

*P < 0.05; **P < 0.01; *χ* ^2^ test.

**Figure 1:**
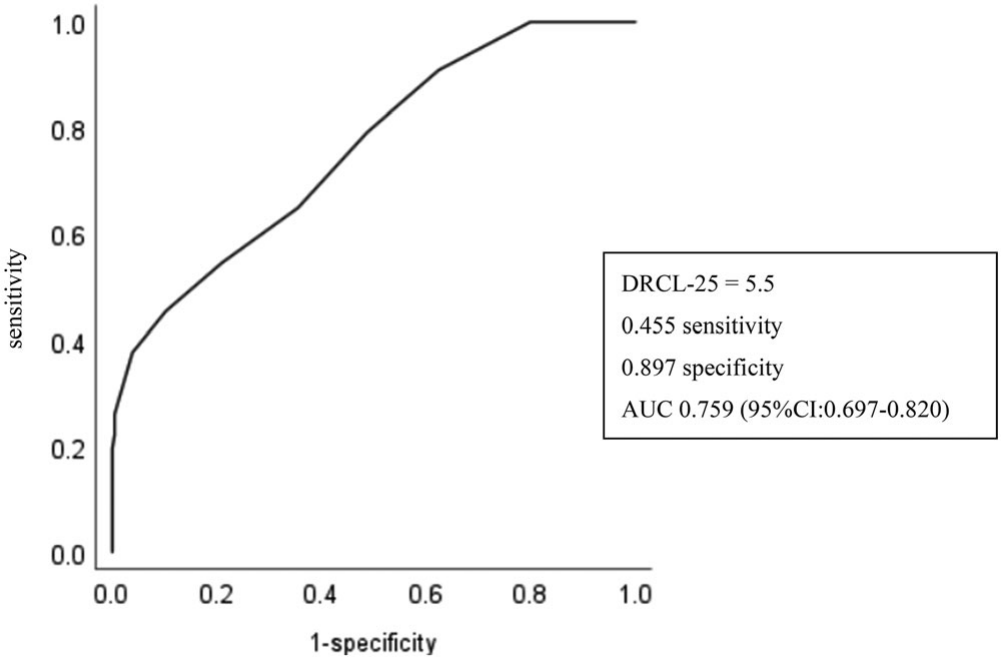
Receiver operating characteristic (ROC) concerning Driving Risk Checklist‐25 (DRCL‐25) for elderly residents in the community.

Finally, Figure 1 illustrates the ROC curve analysis results, which were used to calculate the cutoff value for MCI differentiation based on the DRCL‐25. The AUC for the DRCL‐25 was 0.759 (95% confidence interval: 0.697–0.820, *P* < 0.01). The cutoff value was determined to be 5.5, at which sensitivity was 45.5% and specificity was 89.7%.

## Discussion

### Prevalence of MCI in the study population

This study found that 75.2% of the participants were classified as MCI‐negative, while 24.8% were MCI‐positive (Table 1). Compared with a recent global meta‐analysis reporting an MCI prevalence of 15.56%, this study's population demonstrated a higher proportion of MCI.^14^ This discrepancy may be attributed to regional or sampling factors, warranting further investigation.

### Attributes associated with MCI and driving risk

The analysis of basic attributes revealed that older age, polypharmacy, and lower educational attainment were significantly associated with MCI (Table 1). Polypharmacy, particularly the use of medications with anticholinergic effects, has been linked to memory and attention impairments, further exacerbating driving difficulties in older adults.^15^ Moreover, educational attainment also emerged as a protective factor, likely owing to its association with cognitive reserve. According to Stern's cognitive reserve hypothesis, higher educational levels enhance neural efficiency and resilience, delaying the onset of cognitive decline.^2^ Our findings are consistent with this hypothesis and support previous research.

### Physical assessments and their correlation with MCI

Physical measures, including gait speed and grip strength, were also evaluated in this study (Table 2). Slower gait speed was significantly associated with MCI, supporting the concept of motor–cognitive coupling, wherein declines in motor function reflect underlying neural degeneration.^4^ Reduced grip strength, often indicative of sarcopenia, has similarly been linked to cognitive decline, highlighting the potential of physical fitness assessments as supplementary tools for identifying at‐risk individuals.^5^ Incorporating objective measures of physical performance, such as the timed‐up‐and‐go (TUG) test, into the DRCL‐25 could enhance its predictive accuracy, as the TUG test has been validated as a predictor of fall risk and cognitive impairment.^16^

### Driving‐specific cognitive deficits in MCI

MCI‐positive participants exhibited notable deficits in attention, judgment, and visuospatial coordination, particularly in tasks such as parking, lane changes, and making turns (Table 3). These tasks demand rapid decision‐making, sustained attention, and visuospatial processing, all of which are impaired in MCI.^17^ Additionally, executive function deficits in MCI hinder the ability to anticipate and respond to dynamic traffic situations, increasing error rates. Research by Rizzo *et al*. underscores that individuals with MCI often fail to detect critical hazards, such as sudden pedestrian crossings, owing to deficits in selective and divided attention.^18^ These findings emphasize the need to incorporate specific cognitive domains into the DRCL‐25 to better address real‐world driving challenges faced by older adults with MCI.

### Efficacy and limitations of the DRCL‐25

The ROC curve analysis demonstrated the utility of the DRCL‐25 in distinguishing between MCI‐positive and MCI‐negative individuals, with an AUC of 0.759 (Fig. 1). However, the sensitivity was relatively low (45.5%), suggesting the potential under‐detection of MCI cases. To address this, future research could explore composite screening approaches that integrate neuropsychological tests, such as the TMT and the Clock‐Drawing Test (CDT), which are effective in identifying cognitive deficits related to driving risk. Additionally, incorporating on‐road driving assessments or virtual‐reality‐ (VR)‐based simulations could improve the ecological validity of the DRCL‐25. VR‐based tools have shown promise in capturing real‐time decision‐making and hazard perception, providing a more comprehensive evaluation of driving risk.^19^

### Regional and cultural considerations

The study sample comprised older adults from Akita Prefecture, a rural area in Japan characterized by an aging population and limited public transportation. These contextual factors may influence driving behavior and cognitive function. For example, older adults in rural areas often rely heavily on driving for daily activities, which could motivate them to compensate for cognitive decline.^20^ Cultural differences in driving norms and attitudes toward aging may also impact the generalizability of the DRCL‐25 to other populations. Future research should aim to replicate these findings in diverse settings, including urban areas and different cultural contexts. Cross‐cultural comparisons could help identify universal versus region‐specific predictors of driving risk, enhancing the applicability of the DRCL‐25.

### Implications for public health and policy

The findings of this study have significant public health and policy implications. Screening tools such as the DRCL‐25 could be integrated into routine health check‐ups for older drivers, facilitating the early detection of MCI and timely interventions. High‐risk individuals could be referred to driving rehabilitation programs or supported in transitioning to alternative transportation options, thereby reducing the risk of traffic accidents while maintaining mobility and independence.

### Future directions

Several future research directions emerge from this study. Longitudinal studies are needed to establish causal relationships between cognitive decline and driving risk. In addition, using subjective outcome measures, especially considering that participants with MCI may have difficulty accurately self‐evaluating their own cognitive and functional status, may introduce bias. Future studies should consider incorporating objective assessments and caregiver reports to complement subjective assessments and increase the validity of findings. Additionally, exploring biomarkers such as amyloid‐beta levels or structural MRI findings could provide objective indices of MCI, complementing the DRCL‐25.^21^ Technological advancements, such as AI‐driven analysis of driving behaviors, hold promise for developing more sophisticated screening tools. For instance, in‐vehicle monitoring systems could continuously assess cognitive and physical performance during driving, offering real‐time feedback to drivers and caregivers.^22^ Expanding the applicability of DRCL‐25 to broader populations and further refining its practicality should be prioritized in future research.

## Conclusion

This study assessed driving risk and MCI among older adults, revealing significant differences in driving risk, physical function, and cognitive function between MCI‐positive and MCI‐negative groups. The identified cutoff score of 5.5 on the DRCL‐25 highlights its potential as an effective tool for evaluating driving risk and enhancing traffic safety among older drivers.

Future studies should focus on expanding the applicability of the DRCL‐25 to diverse populations and refining its design to improve usability and precision. Such advancements could further contribute to maintaining mobility and independence while addressing public safety concerns in aging societies.

## Disclosure statement

The authors declare no conflict of interest.

## Supporting information


**Table S1.** Driving Risk Checklist‐25.

## Data Availability

The data that support the findings of this study are available on request from the corresponding author. The data are not publicly available due to privacy or ethical restrictions.
