# Supplementary material for: Characteristics of Driving Risk Checklist‐25 among community‐dwelling older adults with mild cognitive impairment
Source: Geriatr Gerontol Int. 2025 Aug 13;25(9):1194–9. doi: 10.1111/ggi.70127 (PMC12439236; doi:10.1111/ggi.70127)
Supplement: Supplementary file 1 — Table S1. Driving Risk Checklist‐25. [file GGI-25-1194-s001.docx]

Supplementary table 1. Driving Risk Checklist-25

| 1 | When turning the steering wheel in parking lots, etc., I feel that more force is required than before. |
| --- | --- |
| 2 | I feel that the timing of braking is slower than before. |
| 3 | When changing lanes or making a right or left turn, they do not look straight ahead with their own eyes, but only at their mirrors. |
| 4 | I often have near-misses while driving. |
| 5 | I don't care much about dirt and scratches on my car anymore, and I don't feel like cleaning it as much as before. |
| 6 | The number of times I drive for pleasure, such as driving, is gradually decreasing. |
| 7 | I forget where I put my keys, driver's license, etc., and have to look for them more often. |
| 8 | Merging and changing lanes on highways and bypasses became difficult. |
| 9 | More often than not, I lose track of where I parked my car in large parking lots. |
| 10 | I can no longer park well within the confines of my home's garage or parking lot. |
| 11 | After driving, I feel more tired than before. |
| 12 | Sometimes I don't understand the meaning of a road sign that I used to understand. |
| 13 | I have a hard time making right turns at large intersections. |
| 14 | I often get a huff when I suddenly notice pedestrians and bicycles when turning right or left. |
| 15 | I tend to forget to use my blinker when turning right or left or changing lanes. |
| 16 | Even on familiar roads, I made more mistakes such as where to turn. |
| 17 | Talking to passengers while driving became a hassle. |
| 18 | I forgot where I was going while driving. |
| 19 | My usual passengers tell me that my driving has become rougher lately. |
| 20 | Several times I have mis-stomped on the brake and the gas pedal. |
| 21 | There have been a few times when I have driven in the opposite direction without knowing it. |
| 22 | I have forgotten to load passengers or luggage and departed. |
| 23 | I have felt faint or almost fainted while driving. |
| 24 | Often has trouble sleeping at night and sleeps poorly. |
| 25 | I often take medications such as sleep aids to help me sleep. |
|  |  |
